# MOBILE TEXT MESSAGE AS AN ADJUNCTIVE STRATEGY TO SUPPORT HOME-BASED EXERCISE IN OLDER ADULTS

**DOI:** 10.1093/geroni/igae098.2562

**Published:** 2024-12-31

**Authors:** Bethany Kidd, Stephen Jennings, Lauren Abbate, Arti Tayade, Katherine Hall

**Affiliations:** VA Puget Sound Healthcare System, Seattle, Washington, United States; Durham Veterans Affairs Healthcare Adminstration, Durham, North Carolina, United States; VA Eastern Colorado Healthcare System, Aurora, Colorado, United States; Puget Sound VA Heathcare System, Seattle, Washington, United States; Veterans Health Administration/Duke University, Durham, North Carolina, United States

## Abstract

Lack of physical activity is associated with increased comorbidities, falls, frailty and healthcare usage. Several clinical and community-based exercise programs exist to provide guided exercise to older adults, however, staffing and space allotment often create barriers to providing sufficient opportunities. Puget Sound Gerofit sessions are held both in person and virtually. Both classes take place twice a week for 45 minutes. The Gerofit program in Seattle Washington implemented a structured text-based messaging protocol to provide additional exercise opportunities in support of meeting public health guidelines for physical activity. Twenty-six veterans were enrolled using mobile test messaging to provide an additional day of exercise. These veterans were texted 1x a week an automated message asking if they would like to exercise. Positive responses were sent a randomized link from a digital library of pre-recorded exercise videos featuring Gerofit instructors. Follow-up messages were sent to quantify minutes of activity participation. Twenty-six Veterans were messaged, over an average of 24 weeks. Participating veterans were on average 73.7 years old, predominately male (87.0%), and White race (82.6%). Of the 634 messages sent, 145 responses were gathered (22.8%). Veterans who engaged positively (61.5%) in exercise reported an average activity of 33.5 minutes/day. Participating older adults reported high satisfaction with the text-based protocol. Supplementation of a virtual group exercise program is feasible with text messaging and on-demand pre-recorded videos. This format allows a flexible option for participants as well as providing a lower resource option for exercise programs.

